# The central role of radiotherapy in remodeling the tumor immune microenvironment: mechanisms and therapeutic implications

**DOI:** 10.3389/fcell.2026.1868063

**Published:** 2026-06-10

**Authors:** Zhipan Yi, Ning Chen, Xiulin Jiang, Xia Hu

**Affiliations:** 1 Shaoyang Hospital for Maternal and Child Healthcare, Shaoyang, Hunan, China; 2 College of Life Science, University of Chinese Academy of Sciences, Beijing, China

**Keywords:** CD8^+^ T cells, emerging radiotherapy, immune checkpoint inhibitors, immunosuppression, radioresistance, radiotherapy, radiotherapy combination therapy, tumor immune microenvironment

## Abstract

Radiotherapy is an essential component of multidisciplinary cancer treatment. Its role has expanded from conventional local tumor eradication to active regulation of the tumor immune microenvironment. In recent years, emerging radiotherapy strategies, including FLASH radiotherapy, boron neutron capture therapy, lattice radiotherapy, spatially fractionated radiation therapy, precision particle therapy, immunomodulatory stereotactic body radiotherapy, and immune-optimized carbon ion therapy, have provided new opportunities to improve tumor control, reduce normal tissue toxicity, and overcome radioresistance. Radiotherapy can induce DNA damage and immunogenic cell death, promote tumor antigen release, enhance dendritic cell maturation and antigen cross-presentation, and increase CD8^+^ T-cell infiltration and antitumor immunity through the cGAS-STING type I interferon pathway. However, radiotherapy may also trigger immunosuppressive feedback, including the accumulation of myeloid-derived suppressor cells, tumor-associated macrophages, and regulatory T cells, as well as the upregulation of immune checkpoint molecules such as programmed death-ligand 1 (PD-L1). These changes may limit antitumor immune responses and contribute to radioresistance. Combining radiotherapy with immune checkpoint inhibitors can amplify antitumor immunity, but therapeutic efficacy is influenced by dose fractionation, treatment timing, tumor type, and baseline immune status. This mini review summarizes emerging radiotherapy strategies and their regulatory effects on the tumor immune microenvironment, and discusses the mechanistic basis, current challenges, and future directions of radiotherapy combined with immunotherapy.

## 1 Introduction

Radiotherapy (RT) is one of the most important local treatment modalities in cancer care (Giraud et al., 2025). It is widely used as definitive, adjuvant, and palliative therapy for diverse solid tumors (Giraud et al., 2025). Traditionally, RT has been considered to control tumors mainly by directly killing cancer cells through DNA double-strand breaks, oxidative stress, cell cycle arrest, and mitotic catastrophe (Gao et al., 2020). However, with advances in tumor immunology and radiation biology, RT is no longer viewed simply as a local cytotoxic treatment. Increasing evidence indicates that RT can profoundly reshape the tumor immune microenvironment (TIME) and connect local tumor control with systemic antitumor immunity (Gao et al., 2020).

Radiation-induced tumor cell death can be accompanied by the release of tumor-associated antigens and neoantigens (Jiao et al., 2022). It can also promote the exposure or release of damage-associated molecular patterns, such as ATP, HMGB1, and calreticulin (Jiao et al., 2022). These signals enhance antigen uptake, maturation, and cross-presentation by dendritic cells. RT can also activate innate immune pathways, including the cGAS-STING type I interferon axis, leading to the production of inflammatory cytokines and chemokines (Jiao et al., 2022). These events further support the recruitment, activation, and tumor infiltration of CD8^+^ T cells. Through these mechanisms, RT may convert poorly inflamed “cold” tumors into more immune-responsive “hot” tumors (Liu T. et al., 2023). This provides a strong biological rationale for combining RT with immune checkpoint inhibitors (ICIs). In particular, in the context of anti-PD-1/PD-L1 or anti-cytotoxic T-lymphocyte-associated protein 4 (CTLA-4) therapy, RT can provide antigenic and inflammatory signals to enhance T-cell responses, whereas ICIs can release T cells from inhibitory signaling and sustain radiation-induced antitumor immunity (Liu T. et al., 2023). CD8^+^ T cells are central effector cells in RT-induced antitumor immune responses. Several studies have shown that CD8^+^ T-cell infiltration, activation, and cytotoxic function after RT are closely associated with treatment response (Th et al., 2024). For example, in hepatocellular carcinoma models, low-dose radiotherapy promoted the migration of stem-like progenitor exhausted CD8^+^ T cells from draining lymph nodes into tumors through the CXCL10/CXCR3 axis. This process enhanced the therapeutic efficacy of dual PD-L1 and VEGFA blockade (Li S. et al., 2023). However, CD8^+^ T-cell-mediated immune activation is not always sustained. After RT or concurrent chemoradiotherapy, tumor cells and the surrounding immune microenvironment may drive CD8^+^ T-cell senescence, exhaustion, or dysfunction, thereby weakening antitumor immunity and promoting recurrence. In cervical cancer, ACKR2^+^ treatment-resistant tumor cells were shown to induce CD8^+^ T-cell senescence through TGF-β after concurrent chemoradiotherapy (Dai et al., 2024). In a radioresistant non-small cell lung cancer model, RT activated antitumor immunity at an early stage, but the TIME later shifted toward an immunosuppressive state with increased CD8^+^ T-cell exhaustion (Zhang Y. et al., 2023).

In addition to activating antitumor immunity, RT can induce immunosuppressive feedback, which is a major barrier to durable therapeutic benefit and an important contributor to radioresistance (Piffkó et al., 2025). After irradiation, immunosuppressive cells such as myeloid-derived suppressor cells (MDSCs), tumor-associated macrophages (TAMs), and regulatory T cells (Tregs) may accumulate within the TIME. These cells can suppress CD8^+^ T-cell function by secreting factors such as TGF-β, IL-10, and VEGF, expressing immune checkpoint molecules such as PD-L1, or depleting nutrients required for T-cell activation (Wang et al., 2023a). Radiotherapy also regulates immune suppression through an m^6^A-dependent YTHDF2–NF-κB circuit in MDSCs. Ionizing radiation activates NF-κB signaling, which induces YTHDF2 expression in MDSCs (Wang et al., 2023a). Upregulated YTHDF2 then recognizes m^6^A-modified transcripts encoding negative regulators of NF-κB signaling and promotes their degradation. As a result, inhibitory control of NF-κB is weakened, leading to sustained NF-κB activation (Wang et al., 2023a). This positive feedback loop drives MDSC expansion, tumor infiltration, and immunosuppressive function, thereby attenuating antitumor immunity and contributing to radioresistance. Conversely, myeloid-specific Ythdf2 deficiency or pharmacological YTHDF2 inhibition disrupts this circuit, reduces MDSC-mediated immunosuppression, enhances antitumor immune responses, and improves the efficacy of radiotherapy combined with anti-PD-L1 therapy (Wang et al., 2023a). Local RT may also induce systemic expansion of MDSCs and upregulation of PD-L1 on myeloid cells, suggesting that radiation-induced immunosuppression is not restricted to the irradiated site and may influence tumor progression in distant, nonirradiated tissues (Hou et al., 2024). In addition, dynamic changes in Tregs have been linked to the response to radioimmunotherapy in glioblastoma, pancreatic cancer, and esophageal squamous cell carcinoma (Hou et al., 2024).

In recent years, the development of novel RT technologies has further expanded the potential of RT to modulate tumor immunity (Citrin, 2017). Ultra-high dose-rate FLASH radiotherapy (FLASH RT) (Li et al., 2025a), boron neutron capture therapy (BNCT), lattice RT, spatially fractionated RT (SFRT), precision particle therapy, immunomodulatory SBRT, and immune-optimized carbon ion therapy differ in dose rate, linear energy transfer, spatial dose distribution, and biological effects (Citrin, 2017). These emerging strategies may improve tumor dose delivery and reduce normal tissue injury. They may also influence antigen release, inflammatory signaling, immune cell infiltration, and immunosuppressive networks through distinct mechanisms (Citrin, 2017). Therefore, understanding how different RT modalities shape the TIME is important for optimizing dose fractionation, selecting the timing of immunotherapy combinations, overcoming radioresistance, and advancing personalized treatmen (Citrin, 2017).

This mini review focuses on the interaction between RT and the TIME. We first summarize representative emerging RT strategies and their main characteristics. We then discuss how RT enhances antitumor immunity through CD8^+^ T cells and how it can also promote radioresistance through immunosuppressive cells, including MDSCs, TAMs, and Tregs. Next, we review the major mechanisms by which RT improves the efficacy of ICIs. Finally, we discuss future directions in mechanistic studies, combination treatment design, spatial immune profiling, and predictive biomarker development. By integrating these topics, this review aims to clarify the biological basis for the transition of RT from a local treatment modality to an immune-modulating strategy, and to provide a rationale for designing next-generation combinations of RT and immunotherapy.

## 2 Classification of tumor RT

**FIGURE 1:**
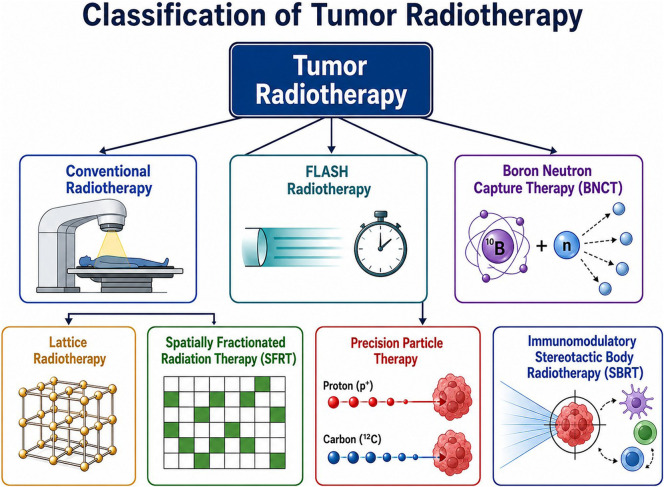
Classification of tumor radiotherapy. The overview of major radiotherapy modalities, including conventional radiotherapy, FLASH radiotherapy, BNCT, lattice radiotherapy, SFRT, precision particle therapy, and immunomodulatory SBRT.

RT is a central component of multidisciplinary cancer treatment. Its basic principle is to use ionizing radiation to induce DNA damage, oxidative stress, cell-cycle arrest, and tumor cell death, thereby achieving local tumor control (Citrin, 2017). With advances in radiation physics, imaging, particle accelerators, dosimetry, and tumor immunology, RT has evolved from conventional two-dimensional or three-dimensional conformal irradiation toward more precise, individualized, and biology-guided strategies. Emerging RT modalities are designed not only to improve tumor dose delivery and reduce normal tissue injury, but also to remodel the tumor immune microenvironment by inducing immunogenic cell death, promoting antigen release, enhancing dendritic cell activation, facilitating T-cell infiltration, and improving responses to immune checkpoint blockade (Citrin, 2017). These modalities can be classified according to dose rate, spatial distribution, radiation type, biological selectivity, and immunomodulatory potential. Representative approaches include FLASH RT, boron neutron capture therapy (BNCT), lattice RT, SFRT, precision particle therapy, immunomodulatory SBRT, and immune-optimized carbon ion therapy (Citrin, 2017). These approaches differ in physical dose distribution, spatial irradiation pattern, biological effect, and clinical application. Emerging RT modalities have expanded the therapeutic landscape of cancer treatment and can be classified according to dose delivery, spatial distribution, radiation type, and immunomodulatory potential (Figure 1).

### 2.1 FLASH RT

FLASH RT is characterized by ultra-high dose-rate irradiation, commonly defined as a mean dose rate of approximately 40 Gy/s or higher (Vozenin et al., 2022). Unlike conventional RT, which delivers radiation over minutes, FLASH can deliver the prescribed dose within an extremely short time. Its most notable feature is the potential to reduce radiation-induced normal tissue injury while maintaining tumor control, known as the “FLASH effect” (Vozenin et al., 2022). This effect may involve transient oxygen depletion, altered reactive oxygen species production, reduced inflammation, vascular preservation, and immune microenvironment remodeling (Hughes and Parsons, 2020). FLASH RT may be especially valuable for tumors located near critical organs, including brain tumors, head and neck cancers, lung tumors, and pediatric malignancies, where conventional RT is limited by normal tissue tolerance (Hughes and Parsons, 2020). It may also reduce uncertainties caused by patient motion and organ displacement. However, FLASH remains at an early translational stage. Specialized equipment, accurate dose monitoring, quality control, variable dose-rate parameters, and tissue- or tumor-specific biological responses remain major challenges. Its mechanisms, indications, dose-fractionation strategies, and long-term safety require further clarification (Moreau et al., 2024).

### 2.2 BNCT

BNCT combines biological targeting with physical selectivity. It relies on the administration of boron-10-containing^10B^ compounds that preferentially accumulate in tumor cells, followed by irradiation with low-energy neutrons (Coghi et al., 2023). Neutron capture by boron-10 generates high-linear energy transfer alpha particles and lithium nuclei, which have a short path length close to the diameter of a single cell. Thus, BNCT can theoretically achieve cell-level precision by killing boron-containing tumor cells while sparing adjacent normal tissues (Coghi et al., 2023). BNCT may be useful for infiltrative, poorly defined, recurrent, or radioresistant tumors, such as recurrent head and neck cancer, malignant glioma, and melanoma (Coghi et al., 2023). Its advantages include high tumor selectivity, high-LET cytotoxicity, reduced surrounding tissue injury, and increasing feasibility through accelerator-based neutron sources (Takahara et al., 2022). However, treatment efficacy depends on selective and homogeneous boron accumulation. Uneven intratumoral boron distribution, normal tissue uptake, neutron beam quality, dose calculation, and limited boron carriers remain key barriers (Takahara et al., 2022).

### 2.3 Lattice RT

Lattice RT is a spatially nonuniform dose delivery technique mainly used for bulky tumors. It creates multiple high-dose “vertices” within the tumor while maintaining lower-dose regions between them, producing a three-dimensional lattice-like dose distribution (Wu et al., 2020). This approach allows local dose escalation in large tumors while maintaining acceptable toxicity. Its biological effects may involve direct tumor cell killing, vascular injury, cytokine release, immunogenic cell death, bystander effects, and immune activation (Np et al., 2024). High-dose regions may promote antigen release and danger signaling, whereas low-dose regions may preserve vascular structures and immune cell function, supporting immune infiltration (Np et al., 2024). SFRT is a broader group of techniques that deliver radiation in spatially heterogeneous patterns, including grid RT, lattice RT, and microbeam RT (Prezado et al., 2024). By combining high-dose and low-dose regions within the same tumor, SFRT may induce strong local cytotoxicity while preserving tissue architecture and immune activity in selected areas (Prezado et al., 2024). It is particularly attractive for bulky, recurrent, or radioresistant tumors when uniform dose escalation is unsafe (Li et al., 2024). However, both lattice RT and SFRT lack standardization regarding vertex size, spacing, dose, fractionation, treatment integration, and biological response modeling. Prospective trials and improved dosimetric-biological models are needed (Li et al., 2024).

### 2.4 Precision particle therapy

Precision particle therapy mainly includes proton therapy and carbon ion therapy. Charged particles deposit energy with a characteristic Bragg peak, allowing high-dose delivery to the tumor with rapid dose fall-off beyond the target (Schaub et al., 2020). Proton therapy is valuable for pediatric tumors, skull base tumors, ocular tumors, head and neck cancers, mediastinal tumors, and lesions adjacent to critical organs, because it can reduce normal tissue exposure and late toxicity (Schaub et al., 2020). Carbon ion therapy combines precise dose distribution with high LET and higher relative biological effectiveness. It induces complex DNA double-strand breaks that are difficult to repair and may be effective against hypoxic, slow-growing, or radioresistant tumors (Verginadis et al., 2025). Carbon ions may also enhance tumor immunogenicity by promoting immunogenic cell death, antigen presentation, and immune microenvironment remodeling (Verginadis et al., 2025). Nevertheless, particle therapy is limited by high cost, restricted access, sensitivity to anatomical changes, and incomplete evidence for some indications. Carbon ion therapy remains less widely available, and optimal combination strategies require further study (Verginadis et al., 2025).

### 2.5 Immunomodulatory SBRT

SBRT delivers highly precise, high-dose radiation to extracranial tumors in a small number of fractions (Kothari et al., 2017). Beyond local ablation, SBRT can promote tumor antigen release, dendritic cell cross-presentation, cGAS–STING type I interferon signaling, and CD8^+^ T-cell-mediated immunity. Immunomodulatory SBRT emphasizes radiation regimens designed to optimize immune activation rather than simply maximize local tumor killing (Kothari et al., 2017). Appropriate fractionation may favor immunogenic cell death and antigen presentation, whereas excessively high single doses may induce TREX1-mediated degradation of cytosolic DNA and weaken type I interferon signaling (Kothari et al., 2017). SBRT is clinically useful in oligometastatic disease and multiple tumor types, and it is well suited for combination with immune checkpoint inhibitors, targeted therapy, anti-angiogenic agents, or cell-based therapy. However, high-dose irradiation may also promote PD-L1 expression, Treg or MDSC recruitment, and T-cell exhaustion. Therefore, its immune effects depend on dose, fractionation, tumor type, baseline immune status, and combination strategy (Kothari et al., 2017).

### 2.6 Immune-optimized carbon ion therapy

Immune-optimized carbon ion therapy represents a next-generation particle therapy strategy that integrates physical precision with tumor biology and immunology (Mairani et al., 2022). Carbon ions can induce complex DNA damage, mitochondrial stress, immunogenic cell death, and inflammatory signaling, thereby enhancing antigen release, antigen presentation, T-cell infiltration, and responses of poorly immunogenic tumors to immunotherapy (Mairani et al., 2022). Future particle therapy may further incorporate LET optimization, adaptive planning, multimodal imaging, and combination with immune checkpoint inhibitors, STING agonists, PARP inhibitors, anti-angiogenic agents, or other radiosensitizers (Chi et al., 2022). However, clinical application remains limited by complex physical and immune variables, uncertain relationships among LET, relative biological effectiveness, and immune activation, high facility costs, limited access, and small clinical cohorts. Predictive biomarkers, such as tumor mutational burden, DNA repair defects, interferon signaling, PD-L1 expression, T-cell infiltration, and myeloid immunosuppression, are needed to guide patient selection (Chi et al., 2022).

### 2.7 RT technique selection and immune effects: differential roles of FLASH and SFRT in immunogenic cell death, cGAS–STING activation, and immune-cell recruitment

Different RT techniques may induce distinct immune consequences, suggesting that radiation delivery should be considered not only as a physical dose-distribution strategy but also as an immune-modulating variable. FLASH RT and SFRT represent two emerging approaches with potentially different immunological profiles (Huang et al., 2025). FLASH RT delivers radiation within an extremely short time window and may reduce normal-tissue toxicity while preserving radiosensitive immune cells, including circulating lymphocytes and tissue-resident immune populations (Hughes and Parsons, 2020). This immune-preserving effect may be particularly valuable when RT is combined with immune checkpoint inhibitors, because excessive lymphocyte depletion or normal-tissue inflammation can compromise systemic antitumor immunity (Hughes and Parsons, 2020). However, because FLASH irradiation may alter oxygen consumption, reactive oxygen species production, and DNA damage kinetics, its capacity to induce immunogenic cell death and activate the cGAS–STING pathway may differ from that of conventional dose-rate irradiation (Hughes and Parsons, 2020). Therefore, FLASH may be especially suitable for clinical settings in which immune preservation, toxicity reduction, and safe combination with immunotherapy are major priorities. In contrast, SFRT creates a highly heterogeneous spatial dose distribution, with high-dose “peak” regions and low-dose “valley” regions within the tumor (McMillan et al., 2024). This pattern may generate a unique immune microenvironment (McMillan et al., 2024). High-dose regions can induce robust tumor cell death, release tumor-associated antigens and damage-associated molecular patterns, and activate DNA damage response and cGAS–STING signaling, thereby promoting type I interferon production and dendritic-cell activation (McMillan et al., 2024). Meanwhile, low-dose valley regions may preserve tumor vasculature, stromal structure, and infiltrating immune cells, facilitating antigen presentation, immune-cell trafficking, and subsequent recruitment of CD8^+^ T cells and other effector immune cells (Tardif et al., 2019). Thus, compared with homogeneous high-dose irradiation, SFRT may provide a better balance between local tumor destruction and intratumoral immune preservation. This feature may make SFRT particularly attractive for bulky, hypoxic, or immunologically “cold” tumors, where stronger immune priming is required.

Based on these differences, we propose a “technique selection–immune effect–combination strategy” hypothesis (Chen et al., 2026). FLASH RT may be preferentially selected when the therapeutic goal is to preserve systemic and normal-tissue immune function, reduce treatment-related toxicity, and expand the safety window for combination with immune checkpoint blockade (Liu et al., 2022). Conversely, SFRT may be selected when the goal is to convert poorly inflamed tumors into immunologically active lesions by enhancing immunogenic cell death, cGAS–STING activation, dendritic-cell priming, and CD8^+^ T-cell recruitment (Chen et al., 2026; Liu et al., 2022). Accordingly, FLASH may be more suitable for combination with PD-1/PD-L1 or CTLA-4 blockade in patients requiring immune preservation (Li et al., 2025b), whereas SFRT may be rationally combined with immune checkpoint inhibitors, STING agonists, dendritic-cell–activating therapies, or myeloid-targeting strategies to amplify local and systemic antitumor immunity (Chen et al., 2026; Liu et al., 2022). Future studies should directly compare FLASH, SFRT, and conventional RT using immune-focused endpoints, including calreticulin exposure, HMGB1 and ATP release, cytosolic DNA accumulation, cGAS–STING–TBK1–IRF3 activation, type I interferon signaling, dendritic-cell maturation, CD8^+^ T-cell infiltration, Treg/MDSC dynamics, and abscopal responses (Chen et al., 2026). Such analyses will help define how specific radiation techniques can be rationally matched with immunotherapeutic partners to maximize antitumor immunity while minimizing radiation-induced immune suppression and tissue injury.

## 3 RT remodels the tumor immune microenvironment

**FIGURE 2:**
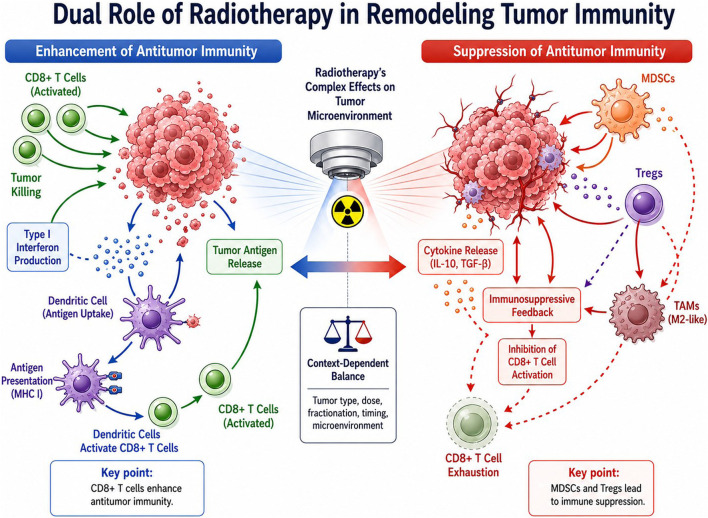
Dual role of radiotherapy in remodeling tumor immunity. Radiotherapy can enhance antitumor immunity by promoting tumor antigen release, type I interferon production, dendritic cell activation, and CD8^+^ T-cell responses, while also inducing immunosuppressive feedback through MDSCs, Tregs, TAMs, and T-cell exhaustion.

RT is not only a local treatment that kills tumor cells. It is also an immune-modulating intervention that can markedly reshape the tumor immune microenvironment (Liu S. et al., 2023). Traditionally, RT has been thought to inhibit tumor growth mainly by inducing DNA double-strand breaks, chromosomal damage, cell cycle arrest, and cell death. However, growing evidence shows that tumor cell death after irradiation is not simply a passive clearance process. It can be accompanied by tumor antigen release, exposure of damage-associated molecular patterns, type I interferon production, dendritic cell activation, and T-cell recruitment (Liu S. et al., 2023). These events can initiate local and even systemic antitumor immune responses. Under favorable conditions, RT may convert the irradiated tumor into an “*in situ* vaccine.” By releasing tumor-associated antigens and neoantigens, RT promotes antigen uptake, processing, and presentation by antigen-presenting cells (Liu S. et al., 2023). This process further activates cytotoxic CD8^+^ T cells and strengthens antitumor immunity. However, the immune effects of RT are bidirectional. On one hand, RT can enhance antitumor immune responses. On the other hand, it may induce immune checkpoint upregulation and promote the infiltration of immunosuppressive cells, including Tregs, MDSCs, and TAMs. These changes may create a new immunosuppressive state, limit therapeutic efficacy, and contribute to radioresistance. Therefore, understanding how RT remodels the tumor immune microenvironment is important for optimizing dose fractionation, improving radioimmunotherapy, and overcoming treatment resistance. RT reshapes the tumor immune microenvironment in a context-dependent manner, exerting both immune-activating and immunosuppressive effects (Figure 2).

### 3.1 CD8^+^ T Cells are essential for RT-Enhanced antitumor immunity

CD8^+^ T cells are among the most important effector cells in antitumor immunity. They recognize tumor antigens presented by MHC class I molecules on tumor cells and directly kill malignant cells by releasing perforin, granzyme B, IFN-γ, and TNF-α. RT-induced antitumor immunity largely depends on the activation, expansion, and tumor infiltration of CD8^+^ T cells (Yu et al., 2021). After irradiation, tumor cells undergo DNA damage and immunogenic cell death. This process releases tumor antigens and immune-stimulatory signals, including ATP, HMGB1, and calreticulin (Yu et al., 2021). These signals promote dendritic cell maturation and enhance antigen uptake and cross-presentation. Mature dendritic cells then migrate to draining lymph nodes, where they activate naïve CD8^+^ T cells and drive their differentiation into cytotoxic effector cells. Activated CD8^+^ T cells subsequently traffic back to the tumor site and eliminate residual tumor cells, thereby amplifying the therapeutic effect of RT.For example, in hepatocellular carcinoma models, low-dose RT (LDRT) enhanced the antitumor efficacy of atezolizumab plus bevacizumab (Herrera et al., 2022). This effect was mainly mediated by CD8^+^ T cells. Mechanistically, LDRT promoted the migration of stem-like progenitor exhausted CD8^+^ T cells from draining lymph nodes into tumors through the CXCL10/CXCR3 axis. These cells then gave rise to effector and cytotoxic CD8^+^ Tex cells, increasing tumor sensitivity to dual PD-L1 and VEGFA blockade (Li S. et al., 2023). In cervical cancer, however, concurrent chemoRT (CCRT) was associated with a different CD8^+^ T-cell outcome. Residual ACKR2^+^ therapy-resistant tumor cells produced TGF-β and induced CD8^+^ T-cell senescence, thereby weakening antitumor immunity and promoting recurrence. Clinical data also linked high ACKR2 expression and CD8^+^ T-cell senescence with recurrence after CCRT and poor prognosis (Dai et al., 2024). In a radioresistant non-small cell lung cancer model, RT activated innate and adaptive immune responses at an early stage. At later stages, however, the tumor immune microenvironment shifted toward immunosuppression and was accompanied by CD8^+^ T-cell exhaustion. Cluster of differentiation 39 (CD39) inhibition combined with RT reduced exhausted CD8^+^ T cells, whereas VISTA blockade plus RT decreased immunosuppressive myeloid cells. These findings suggest that targeting T-cell exhaustion and suppressive immune pathways may help overcome radioresistance (Zhang Y. et al., 2023).CD8^+^ T cells are also closely related to the abscopal effect induced by RT. The abscopal effect refers to regression of distant, nonirradiated tumor lesions after local irradiation. Although this phenomenon is uncommon in clinical practice, it is considered an important sign of systemic antitumor immune activation. Radiation-induced antigen release may activate tumor-specific CD8^+^ T cells throughout the body, enabling them to attack tumor cells outside the irradiated field. Therefore, combining RT with immune checkpoint inhibitors, especially anti-PD-1/PD-L1 or anti-CTLA-4 therapy, is viewed as a promising strategy to enhance CD8^+^ T-cell function and broaden systemic antitumor responses.Nevertheless, CD8^+^ T-cell-mediated immune activation is not always durable. After RT, immune checkpoint molecules such as PD-L1 and CTLA-4 may be upregulated in the tumor microenvironment (Zhang Y. et al., 2023). At the same time, immunosuppressive cells, including Tregs, MDSCs, and TAMs, may accumulate. These changes can drive CD8^+^ T-cell exhaustion, which is characterized by reduced cytotoxicity, decreased cytokine production, and impaired proliferative capacity. Maintaining CD8^+^ T-cell activity, improving tumor infiltration, and preventing functional exhaustion after RT are therefore key challenges for improving radioimmunotherapy.

### 3.2 RT regulates MDSCs

MDSCs are immature myeloid cells derived from bone marrow progenitors. In cancer, chronic inflammation, and infection, MDSCs can expand abnormally and accumulate in peripheral blood, lymphoid organs, and tumor tissues (Kho et al., 2021). Based on phenotype and function, MDSCs are commonly divided into monocytic MDSCs and polymorphonuclear MDSCs (Li et al., 2021). Both subsets have strong immunosuppressive capacity, although their mechanisms and differentiation trajectories may differ. RT can induce MDSC expansion and immunosuppressive activity, thereby limiting antitumor immunity and promoting radioresistance (Kho et al., 2021). One study showed that ionizing radiation increased YTHDF2 expression in MDSCs and established an IR-YTHDF2-NF-κB positive feedback loop. This circuit enhanced MDSC infiltration, differentiation, and suppressive function. Myeloid-specific deletion or pharmacological inhibition of YTHDF2 reduced MDSC-mediated immunosuppression and improved the efficacy of RT and RT combined with anti-PD-L1 therapy (Wang et al., 2023a). Local irradiation may also generate systemic immunosuppressive effects. Analyses of patient peripheral blood and murine models showed that local RT increased systemic MDSC levels and upregulated PD-L1 expression on dendritic cells and myeloid cells. These effects may facilitate metastatic spread in distant, nonirradiated tissues. This process was associated with increased CXCL10 signaling, whereas inhibition of PD-L1 or MDSCs reduced the risk of RT-induced metastasis (Hou et al., 2024). Targeting MDSC-associated immune checkpoints may further enhance the benefit of RT. A CD24/Siglec-10 blocking peptide, CSBP, was shown to block not only CD24/Siglec-10 but also PD-1/PD-L1 interactions. CSBP promoted tumor cell phagocytosis by macrophages and monocytic MDSCs, leading to further activation of CD8^+^ T cells. When combined with RT, CSBP synergistically inhibited tumor growth and improved the immune microenvironment in both anti-PD-1-responsive and anti-PD-1-resistant tumor models (Shen et al., 2023). In glioblastoma, severe and prolonged lymphopenia after standard chemoRT is closely associated with increased MDSCs. Transcriptomic, flow cytometric, and single-cell analyses of peripheral blood mononuclear cells showed that patients with post-treatment lymphopenia had higher expression of MDSC-related genes and increased circulating MDSCs. Preclinical models further demonstrated a causal role for radiation-induced MDSCs in systemic lymphopenia. Pharmacological inhibition of MDSCs using the arginase-1 inhibitor CB1158 or the phosphodiesterase-5 inhibitor tadalafil reduced radiation-related lymphopenia and improved survival (Ghosh et al., 2023). Within the tumor microenvironment, MDSCs suppress antitumor immunity through multiple mechanisms. They may express high levels of arginase-1 and inducible nitric oxide synthase, deplete L-arginine required for T-cell activation, and produce nitric oxide and reactive oxygen species (Ghosh et al., 2023). They can also secrete IL-10, TGF-β, and prostaglandin E2, thereby inhibiting dendritic cell maturation and T-cell activation. In addition, MDSCs promote Treg expansion, induce T-cell exhaustion, and contribute to angiogenesis, invasion, and metastasis (Li et al., 2021). The accumulation of MDSCs can markedly weaken the immune benefit of RT. Even when RT promotes antigen release and CD8^+^ T-cell recruitment, abundant MDSCs can suppress T-cell function and promote residual disease, recurrence, or distant metastasis (Li et al., 2021). Therefore, targeting MDSCs is an important strategy to improve RT efficacy. Potential approaches include blocking MDSC recruitment, inhibiting their suppressive function, promoting their differentiation into mature myeloid cells, or combining these strategies with immune checkpoint blockade to restore T-cell activity. Targets such as CSF1R, CCR2, CXCR2, STAT3, and arginase-1 may help reverse radiation-induced immunosuppression (Li et al., 2021).

### 3.3 RT regulates TAMs

TAMs are among the most abundant immune cells in the tumor immune microenvironment (Mantovani et al., 2017). They may arise from tissue-resident macrophages or from circulating monocytes and monocytic MDSCs that infiltrate tumors and differentiate locally (Mantovani et al., 2017). TAMs are highly plastic. Their phenotypes and functions can change in response to cytokines, metabolites, hypoxia, and treatment-induced stress within the tumor microenvironment. Historically, macrophages have often been divided into M1 and M2 phenotypes (Mantovani et al., 2017). M1-like macrophages are generally considered pro-inflammatory and antitumorigenic. They can produce IL-12, TNF-α, and nitric oxide, and they can promote antigen presentation and T-cell activation. In contrast, M2-like macrophages are commonly associated with tissue repair, angiogenesis, immunosuppression, and tumor progression (Mantovani et al., 2017). However, single-cell technologies have shown that TAMs cannot be fully described by this simple M1/M2 framework. Instead, they exist across a continuum of functional states. Distinct TAM subsets may contribute to immune suppression, angiogenesis, extracellular matrix remodeling, premetastatic niche formation, and treatment resistance (Li Y. et al., 2023). The mechanisms by which RT-induced TAMs contribute to radioresistance are complex (Zhang H. et al., 2023). TAMs can secrete TGF-β, IL-10, VEGF, CCL2, and other mediators that suppress T-cell activation and promote angiogenesis. They can also clear apoptotic cells, support tissue repair, and remodel the extracellular matrix, helping tumors rebuild a growth-permissive niche after irradiation (Li Y. et al., 2023). In addition, TAMs may express immune checkpoint molecules such as PD-L1 and directly inhibit CD8^+^ T-cell function (Jarosz-Biej et al., 2019). Radiation-induced hypoxia may further drive TAMs toward pro-angiogenic and immunosuppressive states. However, TAMs do not always act as negative regulators in RT responses. Their final impact depends on subset composition, spatial distribution, radiation dose, timing, and combination therapy (Jarosz-Biej et al., 2019). If immunosuppressive TAMs can be reprogrammed toward a pro-inflammatory antitumor state, or if monocyte recruitment into tumors can be blocked, RT efficacy may be improved. Potential strategies include inhibition of the CSF1/CSF1R axis, blockade of CCL2/CCR2-mediated monocyte recruitment, targeting PI3Kγ signaling, activation of CD40, and combination with immune checkpoint inhibitors to enhance T-cell function. These approaches provide important opportunities to reduce radiation-induced immunosuppression (Zhang H. et al., 2023).

### 3.4 RT regulates tregs

Tregs are a specialized subset of CD4^+^ T cells characterized by high expression of forkhead box P3 (FOXP3) and cluster of differentiation 25 (CD25) and potent immunosuppressive activity (Ji et al., 2020). Tregs are essential for maintaining peripheral immune tolerance and preventing autoimmunity. In tumors, however, they often suppress antitumor immune responses. Increased Treg abundance is frequently associated with impaired T-cell function, poor response to immunotherapy, and unfavorable prognosis. Tregs inhibit antitumor immunity through several mechanisms (Ji et al., 2020). They secrete immunosuppressive cytokines such as IL-10, TGF-β, and IL-35, which suppress CD8^+^ T cells and natural killer cells. They express high levels of CTLA-4, which binds CD80/CD86 on antigen-presenting cells and reduces costimulatory signaling. They also express CD25 and can competitively consume IL-2, thereby limiting effector T-cell expansion (Ji et al., 2020). In addition, Tregs can strengthen immune suppression through adenosine metabolism, granzyme release, and modulation of dendritic cell function. An increase in Tregs can markedly weaken RT-induced antitumor immunity (Ji et al., 2020). Although RT may promote antigen release and CD8^+^ T-cell activation, simultaneous Treg accumulation can limit therapeutic benefit by suppressing antigen presentation, reducing effector T-cell expansion, and promoting T-cell exhaustion (Tadepalli et al., 2025). In the later phase after irradiation, the ratio of Tregs to CD8^+^ T cells may better reflect the immune status of the tumor microenvironment than the number of Tregs alone. A high Treg/CD8^+^ T-cell ratio usually indicates a more immunosuppressive environment and may predict poor durable tumor control (Tadepalli et al., 2025). Therefore, modulating Tregs may enhance the immune effects of RT. In principle, combining RT with anti-CTLA-4 therapy, anti-CCR4 therapy, low-dose cyclophosphamide, or other Treg-targeting strategies may reduce Treg-mediated suppression and improve CD8^+^ T-cell responses. However, Tregs also maintain normal immune homeostasis (Tadepalli et al., 2025). Excessive depletion of Tregs may increase the risk of autoimmunity or inflammatory toxicity. Clinical translation therefore requires careful selection of treatment timing and intensity according to tumor type, Treg infiltration, CD8^+^ T-cell status, and the immunotherapy regimen (Tadepalli et al., 2025).

In glioblastoma models, fractionated RT markedly increased T-cell infiltration into tumors, but it also induced immunosuppressive feedback. Administration of anti-PD-1 therapy at the peak of post-RT T-cell infiltration produced a better survival benefit than concurrent treatment. However, CD103^+^ Tregs accumulated in the tumor microenvironment after therapy and suppressed CD8^+^ T-cell activation, thereby limiting the response to immune checkpoint blockade. Targeting Tregs promoted tertiary lymphoid structure formation, enhanced CD4^+^ and CD8^+^ T-cell frequency and function, and restored the efficacy of radioimmunotherapy (van Hooren et al., 2023). In pancreatic ductal adenocarcinoma, response to RT was associated with changes in IL-2 receptor expression, including increased IL-2Rβ/γ and decreased IL-2Rα. PD1-IL2v, a PD-1-targeted IL-2 variant, enhanced tumor antigen-specific T-cell activation while reducing Treg-mediated suppression. When combined with single-dose RT, PD1-IL2v induced durable expansion of polyfunctional CD8^+^ T cells, increased T-cell stemness, enhanced tumor-specific memory immunity, activated natural killer cells, and reduced Tregs, resulting in improved local and distant antitumor responses (Piper et al., 2023). In neoadjuvant treatment for esophageal squamous cell carcinoma, RT-based regimens reshaped the immune microenvironment more effectively than chemotherapy alone. These regimens increased CD8^+^ T cells, reduced Tregs, and increased the ratio of effector memory to central memory T cells. Immune checkpoint blockade further enhanced natural killer cell activation and cytotoxicity. These findings suggest that RT-based treatment may improve the response to neoadjuvant chemoRT plus immunotherapy by reducing Tregs and strengthening effector immune cells (Han et al., 2023).

## 4 RT and immunotherapy

**FIGURE 3:**
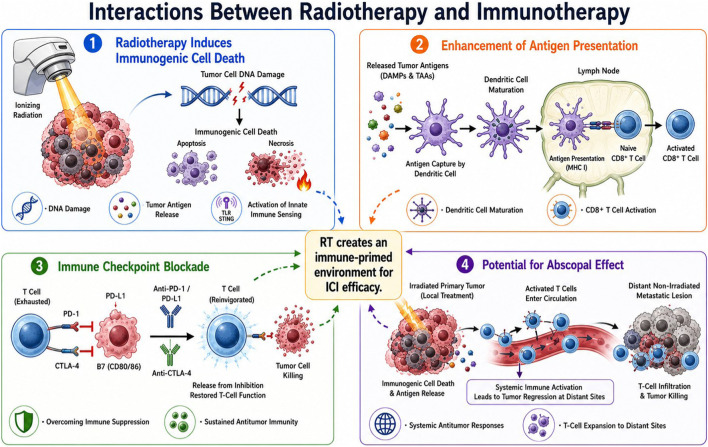
Interactions between radiotherapy and immunotherapy. RT enhances antitumor immunity by inducing immunogenic cell death, antigen release, innate immune activation, and CD8^+^ T-cell priming. ICIs restore T-cell function by blocking PD-1/PD-L1 and CTLA-4 pathways. Their combination may promote systemic immunity and abscopal responses, while clinical optimization depends on treatment timing, dose/fractionation, toxicity control, and predictive biomarkers.

ICIs have reshaped the treatment landscape for diverse malignancies. Antibodies targeting PD-1, PD-L1, and CTLA-4 can relieve T-cell inhibition and restore antitumor immune activity (Bang and Schoenfeld, 2019). However, only a subset of patients achieve durable benefit from ICIs in clinical practice (Bang and Schoenfeld, 2019). Diverse tumors show primary or acquired resistance because of low antigen burden, poor T-cell infiltration, defective antigen presentation, abundant immunosuppressive cells, or persistent immune checkpoint signaling (Bang and Schoenfeld, 2019). Therefore, converting “cold” tumors into “hot” tumors and improving sensitivity to ICIs remain major challenges in cancer immunotherapy. RT has strong potential to enhance ICI efficacy. Traditionally, RT has been viewed as a local treatment that kills tumor cells mainly through DNA damage (Bang and Schoenfeld, 2019). More recent studies indicate that RT also acts as an immune modulator. It can induce immunogenic cell death, promote tumor antigen release, activate innate immune sensing pathways, enhance dendritic cell-mediated antigen presentation, increase CD8^+^ T-cell infiltration, and upregulate immune checkpoint molecules (Bang and Schoenfeld, 2019). These features provide a clear biological basis for combining RT with ICIs: RT supplies antigens and inflammatory signals, whereas ICIs release T cells from inhibitory pathways and help sustain radiation-induced antitumor immunity. To better illustrate the mechanistic basis for combining RT with immunotherapy, we summarized the major immune events induced by RT and their contribution to ICI efficacy in Figure 3. This schematic highlights how RT promotes antigen release, dendritic cell activation, CD8^+^ T-cell responses, immune checkpoint blockade, and potential systemic antitumor effects.

### 4.1 RT promotes tumor antigen release and antigen presentation

One key mechanism by which RT enhances ICI efficacy is the promotion of tumor antigen release and antigen presentation. Radiation can induce DNA damage, apoptosis, necrosis, pyroptosis, and other forms of immunogenic cell death (Russell et al., 2025). These processes release tumor-associated antigens and neoantigens into the tumor microenvironment. At the same time, RT can promote calreticulin exposure, ATP release, and HMGB1 release (Russell et al., 2025). These damage-associated molecular patterns act as danger signals that support dendritic cell recruitment, maturation, and antigen uptake. Dendritic cells play a central role in the synergy between RT and immunotherapy. After irradiation, they can capture tumor-derived antigens and cross-present them through MHC class I pathways to naïve CD8^+^ T cells in draining lymph nodes. This process induces T-cell activation and clonal expansion. Activated tumor-specific CD8^+^ T cells then migrate back to the tumor site and exert cytotoxic effects. When PD-1/PD-L1 or CTLA-4 blockade is added during this process, T-cell priming, expansion, and effector function can be further strengthened. As a result, the magnitude and duration of antitumor immunity may be improved (Russell et al., 2025). In this context, RT may function as an *in situ* vaccine. This effect is particularly important for tumors with weak antigen presentation or poor T-cell infiltration (Russell et al., 2025). By increasing antigen availability and inflammatory signals, RT creates a more favorable immune context for ICIs to work.

### 4.2 RT activates the cGAS-STING-type I interferon pathway

After radiation-induced DNA damage, fragments of double-stranded DNA can enter the cytoplasm and be detected by cGAS. Activated cGAS catalyzes the production of the second messenger cGAMP, which then activates STING and its downstream TBK1/IRF3 signaling axis (Kim and Kim, 2026). This pathway induces type I interferons and multiple inflammatory cytokines and chemokines. Type I interferon signaling promotes dendritic cell maturation, enhances antigen cross-presentation, and supports CD8^+^ T-cell recruitment and effector function (Kim and Kim, 2026). It is therefore a major molecular link between RT-induced DNA damage and antitumor immunity. Activation of the cGAS-STING pathway can also improve the efficacy of ICIs. ICIs mainly act by relieving T-cell inhibition, but they are often insufficient when tumors lack antigen presentation and T-cell infiltration (Kim and Kim, 2026). By activating the cGAS-STING type I interferon axis, RT can strengthen innate immune priming and bridge innate and adaptive immunity, thereby increasing the likelihood of response to PD-1/PD-L1 or CTLA-4 blockade. Importantly, radiation dose and fractionation can affect this pathway (Du et al., 2025). Appropriate fractionated RT may favor cytosolic DNA accumulation and type I interferon production. In contrast, an excessively high single dose may induce DNA exonucleases such as TREX1, leading to cytosolic DNA degradation and weakened cGAS-STING mediated immune activation (Du et al., 2025). Therefore, when designing RT-ICI combinations, it is important to consider not only local tumor ablation, but also the immune consequences of dose and fractionation.

Recent evidence further expands the role of STING beyond its canonical function in cytosolic DNA sensing. Acute ionizing radiation-induced DNA damage can activate PARP1, leading to the synthesis of poly(ADP-ribose) (PAR) (Sun et al., 2025). PAR may directly interact with STING and enhance STING phosphorylation, thereby linking DNA damage repair signaling to STING-dependent cell death (Sun et al., 2025). Unlike the classical cGAS–STING pathway, which is mainly driven by cytosolic DNA and type I interferon responses, this PARP1–PAR–STING axis appears to promote apoptosis through proapoptotic signaling, including PUMA induction and Bax mitochondrial localization (Sun et al., 2025). *In vivo*, STING deficiency or pharmacological reduction of PARP1 activity attenuated radiation-induced intestinal crypt cell death and improved resistance to abdominal irradiation, suggesting that excessive activation of this pathway may contribute to acute radiation injury (Sun et al., 2025). These findings indicate that STING may have dual and context-dependent roles after irradiation: it can enhance antitumor immunity through innate immune activation, but it may also aggravate normal-tissue damage by promoting PAR-dependent apoptosis under acute high-dose radiation exposure (Sun et al., 2025). Therefore, therapeutic modulation of the PARP1–PAR–STING axis may represent a potential strategy to reduce radiation-induced tissue injury while preserving the beneficial immunostimulatory effects of RT.

### 4.3 RT increases CD8^+^ T-Cell infiltration and improves the “cold tumor” state

Poor T-cell infiltration is a major reason why diverse tumors respond poorly to ICIs. These tumors are often described as immunologically “cold (Li Y. et al., 2023).” They usually show weak antigen presentation, low inflammatory cytokine expression, abnormal vasculature, and enrichment of myeloid suppressor cells or Tregs. RT can induce local inflammation and chemokine release, thereby promoting effector T-cell migration into tumor tissues (Li Y. et al., 2023). Loss or downregulation of human leukocyte antigen (HLA)HLA class I molecules is one of the major mechanisms by which tumors escape adaptive immune surveillance and acquire an immunologically “cold” phenotype (Shigematsu et al., 2023; Morris, 2024). HLA class I molecules are essential for presenting tumor-derived antigenic peptides to CD8^+^ cytotoxic T lymphocytes (Cai et al., 2026). Therefore, defects in HLA class I antigen presentation, including genetic loss, transcriptional repression, impaired β2-microglobulin expression, or disruption of antigen-processing components such as TAP1, TAP2, and immunoproteasome subunits, can markedly reduce tumor antigen visibility (Ranjan et al., 2025). As a result, tumor cells become less recognizable to CD8^+^ T cells, leading to weak T-cell infiltration, reduced cytotoxic immune pressure, and poor responsiveness to immune checkpoint blockade (Ranjan et al., 2025). Thus, impaired HLA class I-mediated antigen presentation represents a key molecular basis for immune evasion and cold tumor formation. In colorectal cancer, total loss of MHC class I expression is mainly driven by β2-microglobulin mutations in MSI-positive tumors and antigen-processing machinery defects, especially LMP7 and TAP2 downregulation, in MSI-negative tumors, thereby impairing T-cell recognition and promoting immune evasion (Cabrera et al., 2003).

RT can enhance tumor antigen presentation and promote adaptive antitumor immunity through multiple complementary mechanisms (Zhao et al., 2025; Hsieh et al., 2022). Radiation-induced DNA damage and immunogenic cell death increase the release of tumor-associated antigens and damage-associated molecular patterns, such as HMGB1 and calreticulin exposure, thereby facilitating dendritic cell maturation, phagocytosis, and antigen cross-presentation (Wang et al., 2026). In parallel, radiation-induced cytosolic DNA accumulation activates the cGAS–STING–TBK1–IRF3 axis and type I interferon signaling, which promotes the recruitment and activation of cross-presenting dendritic cells and supports the priming of tumor-specific CD8^+^ T cells (Wang et al., 2023b). Radiation can also induce a “viral mimicry” state, leading to a burst of interferon-stimulated gene expression and the generation of newly synthesized proteins that may serve as additional peptide sources for MHC-I presentation (Amundson et al., 2003). These effects collectively increase tumor antigenicity and help initiate adaptive immune responses within the irradiated tumor microenvironment. Importantly, RT can directly enhance the antigen-processing and presentation machinery in tumor cells. Several studies have shown that ionizing radiation upregulates MHC class I molecules and key antigen-processing components, including β2-microglobulin, TAP1, TAP2, LMP2, LMP7, and other immunoproteasome subunits (Beck et al., 2024; Koukourakis et al., 2023; Dai et al., 2021; Xu et al., 2021). Radiation may also broaden the MHC-associated peptidome and generate radiation-specific peptide repertoires, thereby increasing the visibility of tumor cells to CD8^+^ cytotoxic T lymphocytes (Herrera et al., 2022). In addition, radiation-induced chemokines such as CXCL9, CXCL10, and CXCL16 can recruit effector T cells into the tumor microenvironment (Wang C. L. et al., 2023; Lee et al., 2023). However, increased CD8^+^ T-cell infiltration may also induce adaptive immune resistance, including activation of the PD-1/PD-L1 pathway (Lyu et al., 2025; McLaughlin et al., 2020). Therefore, RT may help convert immunologically “cold” tumors with weak antigen presentation and poor T-cell infiltration into more immune-inflamed tumors, providing a strong mechanistic rationale for combining RT with immune checkpoint blockade (Lyu et al., 2025).

### 4.4 RT upregulates immune checkpoint molecules and provides a rationale for ICI combination

While RT can enhance antitumor immunity, it can also induce the upregulation of immune checkpoint molecules on tumor cells and immune cells, including PD-L1, CTLA-4, TIM-3, LAG-3, and TIGIT (Antonia et al., 2017). This phenomenon can be regarded as adaptive immune resistance after radiation-induced immune activation. In other words, after RT activates T cells and inflammatory responses, the tumor microenvironment may increase checkpoint signaling to limit immune pressure and support immune escape (Antonia et al., 2017). PD-L1 upregulation is one of the most common negative feedback mechanisms after RT. Radiation-induced IFN-γ can promote PD-L1 expression on tumor cells and myeloid cells. Once PD-L1 binds PD-1 on T cells, T-cell receptor signaling is suppressed, cytotoxic molecule release is reduced, and T-cell exhaustion may develop (Zhang et al., 2022). Therefore, increased PD-L1 after RT may indicate immune activation, but it may also become a barrier to durable therapeutic benefit (Zhang et al., 2022). This feature provides a strong rationale for combining RT with ICIs. RT promotes antigen release, T-cell recruitment, and immune activation, whereas ICIs block the suppressive feedback induced by RT. This combination can enhance and prolong antitumor immunity. In tumors where PD-L1 expression is induced by RT, PD-1/PD-L1 blockade may be particularly relevant.

#### 4.4.1 Technique-dependent immune effects of RT

The immunological outcome of RT is determined not only by radiation dose but also by how radiation is delivered. Different delivery strategies may shift the balance between antitumor immune activation and radiation-induced immune suppression. FLASH RT, which uses ultra-high dose rates, may reduce normal-tissue injury and better preserve radiosensitive immune compartments, including circulating lymphocytes and tissue-resident immune cells (Prezado et al., 2024). This feature may help maintain systemic immune competence and provide a safer window for combination with immune checkpoint blockade. By contrast, spatially fractionated RT (SFRT) produces a heterogeneous dose pattern within tumors, with high-dose regions causing intensive tumor-cell damage and low-dose regions preserving parts of the vascular and stromal microenvironment (Prezado et al., 2024). The high-dose regions may enhance immunogenic cell death, tumor antigen release, damage-associated molecular pattern production, and cytosolic DNA accumulation, thereby promoting cGAS–STING activation and type I interferon signaling. Meanwhile, the lower-dose regions may support antigen-presenting cell function, immune-cell trafficking, and recruitment of effector cells such as CD8^+^ T cells (Prezado et al., 2024; Sun et al., 2025). Therefore, FLASH and SFRT may exert different immune-modulating effects and should be matched with distinct therapeutic goals. FLASH may be preferentially used when immune preservation, toxicity reduction, and safe integration with checkpoint inhibitors are priorities, whereas SFRT may be more suitable for bulky, hypoxic, or immune-cold tumors that require stronger local immune priming through immunogenic cell death, cGAS–STING activation, and effector immune-cell recruitment (Sun et al., 2025). This framework supports a more personalized radioimmunotherapy strategy in which dose rate, spatial dose distribution, tumor immune phenotype, and immunotherapy partner are considered together to maximize antitumor immunity while limiting radiation-associated toxicity.

## 5 Future perspectives

With advances in RT technology, tumor immunology, and precision medicine, RT is increasingly recognized not only as a local tumor-killing strategy but also as an immune-modulating approach. Emerging RT modalities, including FLASH RT, BNCT, SFRT, lattice RT, SBRT, proton therapy, and carbon ion therapy, provide new opportunities to improve tumor control, reduce normal tissue toxicity, and overcome radioresistance. However, their effects on tumor immunity are complex and may involve both immune activation and immunosuppressive feedback. Therefore, future studies should focus on clarifying modality-specific immune mechanisms, optimizing combination strategies, and identifying reliable biomarkers for individualized treatment. Personalized Radioimmunotherapy Based on Tumor Immune Subtype and Genomic Features.

### 5.1 Personalized radioimmunotherapy: Technique selection, immune phenotype, and dynamic biomarkers

Future optimization of RT combined with immunotherapy should move beyond a uniform treatment model and instead integrate radiation technique, tumor immune phenotype, genomic features, and dynamic biomarkers. Tumors with a “hot” immune phenotype, characterized by pre-existing CD8^+^ T-cell infiltration, active interferon signaling, PD-L1 expression, and intact antigen presentation, may be more suitable for RT as an immune-amplifying strategy (Gao et al., 2022). In these tumors, moderate hypofractionated RT or SBRT may further enhance antigen release, dendritic-cell activation, and effector T-cell function, while immune checkpoint inhibitors such as anti-PD-1/PD-L1 or anti-CTLA-4 antibodies may sustain antitumor immunity (Gide et al., 2019). Tumors with high TMB or MSI-H/dMMR status may also benefit from this strategy because of increased neoantigen availability and higher sensitivity to checkpoint blockade. In contrast, immunologically “cold” tumors, which often show poor T-cell infiltration, hypoxia, stromal exclusion, defective antigen presentation, or myeloid-dominant immunosuppression, may require RT to act as an immune-priming intervention. In this setting, large-fraction SBRT, spatially fractionated RT, or other immune-stimulatory radiation approaches may be used to induce immunogenic cell death, activate cGAS–STING/type I interferon signaling, promote dendritic-cell maturation, and recruit CD8^+^ T cells into the tumor microenvironment (Zhao et al., 2023). However, because cold tumors may not respond sufficiently to checkpoint blockade alone, rational combinations may include RT with STING agonists, dendritic-cell activators, anti-angiogenic agents, TGF-β inhibitors, or therapies targeting MDSCs and tumor-associated macrophages.

RT technique and fractionation should also be personalized according to the intended immune effect. SBRT or hypofractionated RT may be more suitable when strong local immune priming is required, whereas conventional or moderate fractionation may be preferred when large treatment fields are involved and lymphocyte preservation is important (Zhao et al., 2023). However, there is still no universal consensus on the optimal dose and fractionation pattern for radioimmunotherapy. Excessively high single-fraction doses may induce strong tumor-cell damage but may also promote immune suppression or limit cGAS–STING activation in certain contexts (Zhao et al., 2023). Therefore, future clinical trials should directly compare different radiation techniques and fractionation schedules using immune-focused endpoints.

Importantly, static biomarkers such as PD-L1, TMB, and MSI status are insufficient to fully predict the efficacy of RT combined with immunotherapy (Yang et al., 2024). Dynamic biomarkers should be incorporated to monitor treatment-induced immune remodeling. Peripheral blood immune-cell changes, including absolute lymphocyte counts, CD8^+^ T-cell frequency, neutrophil-to-lymphocyte ratio, Tregs, MDSCs, and exhausted T-cell subsets, may reflect systemic immune activation or suppression during treatment (Yang et al., 2024). ctDNA kinetics may provide an early indicator of tumor burden and therapeutic response, with rapid ctDNA clearance suggesting effective tumor control and persistent or rising ctDNA indicating potential resistance (Leite da et al., 2025). In addition, intratumoral TCR clonality and expansion of tumor-reactive T-cell clones may help determine whether RT successfully induces productive antitumor immune priming (Leite da et al., 2025). Together, these considerations support a biomarker-guided “tumor immune phenotype–genomic feature, RT technique–immunotherapy partner” framework. In this model, hot or TMB-high/MSI-H tumors may benefit from RT as an immune enhancer combined with checkpoint blockade (Liu et al., 2019), whereas cold or immune-excluded tumors may require immune-priming radiation techniques combined with additional agents targeting innate immune activation, vascular normalization, stromal barriers, or suppressive myeloid cells (Liu et al., 2019). Such an integrated strategy may improve patient selection, optimize radiation dose and fractionation, and maximize the therapeutic benefit of radioimmunotherapy while minimizing radiation-induced immune suppression and tissue injury.

**FIGURE 4:**
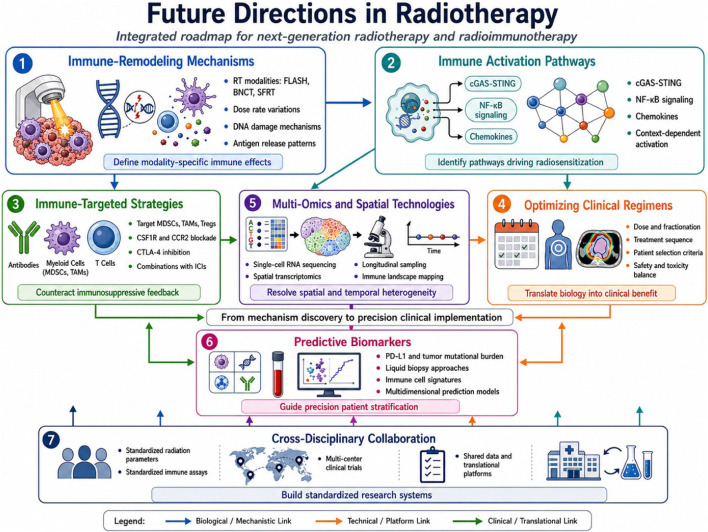
Future directions in radiotherapy and radioimmunotherapy. This roadmap outlines key priorities for advancing radiotherapy-based cancer treatment, including immune-remodeling mechanisms, rational combination strategies, regimen optimization, multi-omics biomarker discovery, and translational collaboration. These efforts may improve patient stratification and support standardized clinical application.

Future work should therefore advance at multiple levels, including basic mechanisms, technical optimization, rational combinations, clinical translation, and biomarker discovery. Based on current challenges and unresolved questions in RT-based immunomodulation, we further propose a roadmap for future research directions in Figure 4. This overview emphasizes the need to integrate mechanistic studies, immune-targeted strategies, multi-omics technologies, biomarker discovery, clinical optimization, and cross-disciplinary collaboration. First, the immune-remodeling effects of different RT modalities need to be systematically defined. Because these approaches differ in dose rate, LET, spatial dose distribution, fractionation, and tissue penetration, they may induce distinct patterns of DNA damage, antigen release, dendritic cell activation, CD8^+^ T-cell infiltration, myeloid cell recruitment, and immune checkpoint expression. Integrating radiation biology, spatial omics, single-cell analysis, and mathematical modeling will help reveal how each modality reshapes the tumor immune microenvironment. Second, future research should identify the key immune pathways that determine radiosensitization or radioresistance. RT can activate cGAS-STING type I interferon signaling, NF-κB pathways, chemokine networks, and immunogenic cell death, thereby promoting antigen presentation and T-cell-mediated tumor clearance. However, excessive or chronic immune activation may also induce inflammation, MDSC recruitment, Treg expansion, TAM polarization, and T-cell exhaustion. Understanding the balance between immune activation and suppression will be essential for designing effective therapeutic regimens. Third, rational combinations of RT with immunotherapy or targeted therapy should be further optimized. The timing, dose, fractionation, irradiation volume, and treatment sequence remain key unresolved issues. Early after RT, enhancing antigen presentation and T-cell priming may be most beneficial, whereas later stages may require blockade of immune checkpoints, myeloid suppression, or T-cell exhaustion. Prospective clinical trials and real-world studies are needed to determine the optimal strategies for different tumor types and immune backgrounds. Finally, predictive biomarkers and standardized research systems are critical for clinical translation. Potential biomarkers include PD-L1 expression, tumor mutational burden, DNA damage repair defects, cGAS-STING activity, type I interferon signatures, CD8^+^ T-cell infiltration, TCR clonality, and the ratios of MDSCs, Tregs, and TAMs. Multi-omics, spatial technologies, radiomics, and liquid biopsy may support dynamic monitoring of immune responses after RT. In addition, standardized reporting of radiation parameters, immune assays, sampling time points, and toxicity assessment will be necessary to compare results across studies and accelerate individualized RT-immunotherapy strategies.

### 5.2 RT combined with Non-ICI immunotherapies

Beyond immune checkpoint inhibitors, RT may also be integrated with other immunotherapeutic strategies, including oncolytic viruses, cancer vaccines, and adoptive cell therapies such as CAR-T, TCR-T, and TIL therapy (Wang et al., 2024). RT can enhance tumor antigen release, promote antigen presentation, remodel the tumor microenvironment, and activate innate immune pathways such as cGAS–STING, thereby providing a rational basis for these combinations (Wang et al., 2024). For example, STING activation has been shown to strengthen TCR-T cell antitumor activity by enhancing antigen presentation, activating STING- and TCR-related signaling, and increasing IFN-γ production, suggesting that innate immune stimulation may improve engineered T-cell recognition of tumors with low antigen expression (Wang et al., 2024). In this context, RT-induced DNA damage and STING activation may synergize with adoptive T-cell therapy by increasing tumor immunogenicity and facilitating effector T-cell function (Wang et al., 2024). Similarly, RT may improve cancer vaccine efficacy by increasing antigen availability and dendritic-cell priming, while oncolytic viruses may cooperate with RT to amplify local inflammation and tumor-specific immune responses (Wang et al., 2024). However, these strategies still face major challenges, including optimal sequencing, radiation dose selection, toxicity control, antigen heterogeneity, T-cell exhaustion, poor immune-cell infiltration, and immunosuppressive myeloid barriers (Wang et al., 2024). Future studies should define how RT can be rationally combined with STING agonists, vaccines, oncolytic viruses, and adoptive cell therapies to convert local tumor damage into durable systemic antitumor immunity.

## 6 Conclusion

In conclusion, RT has evolved from a local cytotoxic modality into a powerful immune-modulating strategy that reshapes the tumor immune microenvironment. By inducing DNA damage, immunogenic cell death, tumor antigen release, dendritic cell activation, cGAS-STING–type I interferon signaling, and CD8^+^ T-cell infiltration, RT can enhance antitumor immunity and improve the efficacy of immune checkpoint inhibitors. However, RT can also promote immunosuppressive feedback through MDSCs, TAMs, Tregs, and immune checkpoint upregulation, thereby contributing to radioresistance. Emerging RT modalities, including FLASH RT, BNCT, SFRT, SBRT, proton therapy, and carbon ion therapy, offer new opportunities to optimize immune activation while reducing toxicity. Future studies should define modality-specific immune mechanisms, refine dose and fractionation strategies, identify predictive biomarkers, and develop personalized radioimmunotherapy combinations to maximize durable antitumor responses while minimizing radiation-induced immune suppression.
